# *Candida *soluble cell wall β-glucan facilitates ovalbumin-induced allergic airway inflammation in mice: Possible role of antigen-presenting cells

**DOI:** 10.1186/1465-9921-10-68

**Published:** 2009-07-21

**Authors:** Ken-ichiro Inoue, Hirohisa Takano, Eiko Koike, Rie Yanagisawa, Toshio Oda, Hiroshi Tamura, Yoshiyuki Adachi, Ken-ichi Ishibashi, Naohito Ohno

**Affiliations:** 1 Environmental Health Sciences Division, National Institute for Environmental Studies, Tsukuba, Ibaraki 305-8506, Japan; 2 Seikagaku Biobusiness Corporation, Tokyo, Japan; 3 Laboratory for Immunopharmacology of Microbial Products, School of Pharmacy, Tokyo University of Pharmacy and Life Science, Japan

## Abstract

**Background:**

Although fungi have been implicated as initiating/deteriorating factors for allergic asthma, their contributing components have not been fully elucidated. We previously isolated soluble β-glucan from *Candida albicans *(CSBG) (Ohno et al., 2007). In the present study, the effects of CSBG exposure on airway immunopathology in the presence or absence of other immunogenic allergen was investigated *in vivo*, and their cellular mechanisms were analyzed both *in vivo *and *in vitro*.

**Methods:**

*In vivo*, ICR mice were divided into 4 experimental groups: vehicle, CSBG (25 μg/animal), ovalbumin (OVA: 2 μg/animal), and CSBG + OVA were repeatedly administered intratracheally. The bronchoalveolar lavage cellular profile, lung histology, levels of cytokines and chemokines in the lung homogenates, the expression pattern of antigen-presenting cell (APC)-related molecules in the lung digests, and serum immunoglobulin values were studied. *In vitro*, the impacts of CSBG (0–12.5 μg/ml) on the phenotype and function of immune cells such as splenocytes and bone marrow-derived dendritic cells (BMDCs) were evaluated in terms of cell proliferation, the surface expression of APC-related molecules, and OVA-mediated T-cell proliferating activity.

**Results:**

*In vivo*, repeated pulmonary exposure to CSBG induced neutrophilic airway inflammation in the absence of OVA, and markedly exacerbated OVA-related eosinophilic airway inflammation with mucus metaplasia in mice, which was concomitant with the amplified lung expression of Th2 cytokines and IL-17A and chemokines related to allergic response. Exposure to CSBG plus OVA increased the number of cells bearing MHC class II with or without CD80 in the lung compared to that of others. *In vitro*, CSBG significantly augmented splenocyte proliferation in the presence or absence of OVA. Further, CSBG increased the expression of APC-related molecules such as CD80, CD86, and DEC205 on BMDCs and amplified OVA-mediated T-cell proliferation through BMDCs.

**Conclusion:**

CSBG potentiates allergic airway inflammation with maladaptive Th immunity, and this potentiation was associated with the enhanced activation of APCs including DC.

## Background

Bronchial asthma has been recognized as a chronic type of allergic airway inflammation with hyperresponsiveness to foreign allergens. Almost all allerologists know that allergy including asthma involves a significant genetic component; however, to explain the rapid rise in the prevalence of asthma and allergic diseases over the past 40 years, we must consider environmental factors and triggers [1]. Thus, understanding the nature of environmental triggers is fundamental in attempts to prevent/reduce allergic diseases, in particular, in the next generation. As previously noted, among environmental triggers/factors, exposure to common aeroallergens, especially perennial inhalable allergens such as house dust mite, companion animal allergens, cockroach allergen, and pollutants, is associated with a significantly increased risk for asthma [2,3]. Fungal spores are also important, since their growth is a common problem in moisture- and water-damaged buildings [3-5]. Given the evidence of the importance of exposure to fungi including molds as an environmental risk factor for asthma, some epidemiological data have been reported on the link between airborne fungi and asthmatic subjects [6,7], e. g. exposure to fungi in indoor air is thought to induce allergic asthma in susceptible subjects [8]. More importantly, fungi may increase allergic reactions to other allergens as adjuvants [8]. However, there is still a shortage of experimental evidence regarding the correlation between fungi and allergic diseases. Furthermore, it remains unknown which components of the fungal organisms are responsible for the induction/adjuvant effects on allergic asthma.

β-glucan of fungi reportedly possesses various biological activities [9,10], being implicated in the development of mycosis. However, the activities of the component have not been appropriately elucidated, because it cannot be solubilized easily in water and alkali solutions, resulting in a difficulty to examine it experimentally [11]. We have succeeded in the extraction of a soluble β-glucan from *C. albicans *(CSBG), recognized to be a major opportunistic pathogen in humans, and analyzed its biological effects [12]. We previously reported its immunopharmacological activities such as the production of proinflammatory cytokines *in vitro *[13] and antitumor activity *in vivo *[14]. Also, we demonstrated that a single pulmonary exposure to CSBG induces lung inflammation characterized by the infiltration of inflammatory leukocytes including eosinophils with an enhanced lung expression of proinflammatory cytokines and chemokines, with a possible link to the activation of signal transducer and activator of transcription (STAT)6 [15], implicating that CSBG can induce/facilitate allergic airway inflammation, in which STAT6 pathway plays an important role. Furthermore, some previous studies have implied that gastrointestinal tract colonization by *Candida *enhances allergic airway responses *in vivo *[16,17] and in humans [18].

The present study explored whether repetitive pulmonary exposure to CSBG induces allergic type airway inflammation and/or potentiates other allergen (ovalbumin: OVA)-induced allergic airway inflammation in mice. Further, we examined the impact of CSBG on immune cells such as splenocytes and bone marrow-derived dendritic cells (BMDCs) *in vitro *to identify cellular mechanisms underlying CSBG's facilitation on allergic inflammation.

## Methods

### Reagents

CSBG was purified using the NaClO-DMSO method from the yeast form of *C. albicans *NBRC1385 cultured on C-limitting medium, as previously described [13]. Briefly, *C. albicans *was oxidized with NaClO solution, and the resulting insoluble part was collected and extensively washed and dialyzed with distilled water. Finally, the insoluble part (NaClO-oxidized candida: OX-CA) was dried by acetone and ether. OX-CA was essentially composed of β-glucan and free from nitrogen based on elemental analysis. CSBG was extracted from OX-CA by dimethyl sulfoxide (DMSO: Sigma Chemical, St. Louis, MO) and precipitated using acetone and ether. Thus, CSBG is a purified preparation of solubilized cell wall β-glucan of *C. albicans*. The structure of CSBG was determined by elemental analysis, β-1,3-glucanase digestion, MALDI-TOF-MASS analysis, and NMR spectroscopy as β-1,3-glucan carrying β-1,6-β segment [13]. The activity of OX-CA (parent material of CSBG) was completely dependent on dectin-1, a β-glucan receptor, by using alveolar macrophage from dectin-1 knockout mice, suggesting that CSBG is a ligand of dectin-1 [19]. CSBG was dissolved in DMSO. The endotoxin activity which was determined using the Limulus Amebocyte Lysate assay (Seikagaku-kogyo, Tokyo, Japan), was lower than the detection limit (0.001 EU/ml) in the vehicle and CSBG.

### Animals

Male ICR mice 6 wk of age (Japan Clea Co., Tokyo, Japan) were used for the *in vivo *studies; mice 11–13 wk of age were used for *in vitro *studies. Mice were housed in an animal facility maintained at 24 to 26°C with 55 to 75% humidity and a 12-h light/dark cycle, and fed a commercial diet (Japan Clea Co.) and given water ad libitum. The studies adhered to the National Institutes of Health guidelines for the experimental use of animals. All animal studies were approved by the Institutional Review Board of the National Institute for Environmental Studies.

### In vivo studies

#### Study protocol

Mice were divided into four experimental groups. The vehicle group received vehicle (phosphate-buffered saline: PBS at pH 7.4 [Invitrogen Co., Carlsbad, CA, USA] containing 2% DMSO) every 2 wk for 4 wk (total: 3 times). The OVA group received 2 μg of OVA (Grade IV: Sigma Chemical: endotoxin activity was lower than the detection limit) dissolved in the same vehicle every 2 wk for 4 wk (total: 3 times) to elicit allergic airway inflammation according to the previous study by our laboratory [20]. The CSBG group received (12.5 or) 25 μg of CSBG suspended in the same vehicle every 2 wk for 4 wk (total: 3 times). This protocol was confirmed to significantly facilitate this type of allergic asthma when intratracheally administered 3 times, since the effect of a 6-times CSBG exposure on the pathology was not different from a 3-times exposure. The CSBG + OVA group received the combined treatment using the same protocol as the OVA and CSBG groups, respectively. In each group, vehicle, CSBG, OVA, or CSBG + OVA was dissolved in 0.1-ml aliquots, and inoculated by the intratracheal route through a polyethylene tube under anesthesia with 4% halothane (Hoechst, Japan, Tokyo, Japan). Twenty-four h after the last intratracheal administration, mice were sacrificed and sera and the lungs were collected for bronchoalveolar lavage (BAL) cellularity, lung histology, lung cytokine profile, and OVA-specific antibody assay. In other experiments, 4 h after the last intratracheal administration, mice were sacrificed and the lungs were collected for FACS analysis.

#### BAL and histological evaluation

BAL and cell counts were conducted as previously described [20-22] (n = 8 in each group). After the BAL procedure, the lungs were removed, snap-frozen in liquid nitrogen, and stored at -80°C for ELISA. Histological evaluation was conducted using hematoxylin and eosin (H&E) staining (International Reagents Co., Kobe, Japan) or periodic acid-Schiff (PAS) (n = 5 in each group), as previously described [21,22].

#### Morphometric analysis of numbers of polymorphonuclear leukocytes (PMNs), mononuclear cells (MONs), and mucus cells

On another occasion, sections were stained with Diff-Quik to quantify the numbers of infiltrated PMNs and MONs. The length of the basement membrane of airways was measured by videomicrometer (Olympus Co., Tokyo, Japan) in each sample slide. The numbers of PMNs and MONs around the airways were counted with a micrometer under oil immersion. The results are expressed as the number of inflammatory cells per millimeter of basement membrane, as described previously [21] (n = 5 in each group). To quantify mucus cells, sections were stained with PAS. The number of mucus cells in the bronchial epithelium was counted using a micrometer. The results are expressed as the number of mucus cells per millimeter of basement membrane, as described previously [21] (n = 5 in each group).

#### Quantification of cytokines in lung tissue homogenates

Frozen lungs were subsequently homogenized, as described previously [21,22]. ELISAs for interleukin (IL)-4 (Amersham, Buckinghamshire, UK), IL-5 (Endogen, Cambridge, MA), IL-13 (R&D systems, Minneapolis, MN), interferon (IFN)-γ (Endogen), IL-17A (BioLegend, Inc., San Diego, CA), granulocyte/macrophage-colony-stimulating factor (GM-CSF), eotaxin, macrophage inflammatory protein (MIP)-1α, and keratinocyte-derived chemoattractant (KC)(R&D systems) in the lung tissue homogenates were conducted according to the manufacturer's instructions (n = 8 in each group).

#### Total IgE and OVA-specific Ig determination

Total IgE, OVA-specific IgG_1_, IgG_2a_, or IgE antibodies was measured by ELISA using sera obtained from blood, as previously described [20,21]. In brief, for total IgE, microplate wells were coated with a rat anti-mouse IgE monoclonal antibody (Yamasa Syoyu Co., Chiba, Japan) and diluted samples were introduced. After incubation, biotinylated rat anti-mouse IgE (BD Biosciences Pharmingen, San Diego, CA) was added to each well. Thereafter, the wells were incubated with 4-methylumbelliferyl-β-galactoside (Sigma Chemical) as the enzyme substrate. The fluorescene intensity was read by a microplate fluorescene reader (Fluoroskan Flow Laboratories, Costa Mesa, CA)(n = 13 in each group). For OVA-specific IgG_1 _and IgG_2a_, microplate wells (Dynatech, Chantilly, VA) were coated with OVA. Diluted samples were added to the microplate and the bound IgG_1 _was detected with biotinylated rabbit anti-mouse IgG_1 _or IgG_2a _(Zymed Laboratories, San Francisco, CA). Color development was achieved using horseradish-peroxidase-conjugated streptavidin (Sigma Chemical) followed by the addition of o-phenylenediamine and H_2_O_2_. Optimal density readings of samples at 492 nm were obtained, and results were expressed in titers calculated based on the titers of the standard serum (n = 13 in each group). OVA-specific IgE was measured using an ELISA kit (Dainippon Sumitomo Pharmaceutical Co., Osaka, Japan) according to the manufacturer's instructions. Subtractive readins at 450 nm was converted to ng/ml using values obtained from standard curves generated with varying concentrations of anti-OVA antibodies (n = 13 in each group).

#### Preparation of lung cells and FACS analysis

Mice were anesthetized with sodium pentobarbital (Dainippon Sumitomo Pharmaceutical Co.) given intraperitoneally (50 mg/kg), and exsanguinated from the cut abdominal aorta and vein. The lung cells were prepared as previously described [20]. Briefly, the lung vessel was perfused with 2 ml of chilled PBS containing 0.2% bovine serum albumin (BSA; Sigma Chemical) and 10 U/ml heparin (Wako Pure Chemical Industries, Ltd., Osaka, Japan) introduced into the right ventricle. The lung tissue was sliced and digested for 90 min at 37°C in 2 ml of PBS containing 0.2% BSA, 0.25 mg/ml collagenase A (Boehringer Mannheim, Mannheim, Germany), and 50 U/ml DNAase I (Sigma Chemical). After teasing the digested tissue and filtration through cotton wool, single cell suspensions were collected. The numbers of viable cells were determined by the trypan blue exclusion method.

For FACS analysis, the following monoclonal antibodies were used: MHC class II molecules: I-A/I-E (2G9, FITC-conjugated, BD Biosciences Pharmingen); co-stimulatory molecules: CD80 (16-10A1, PE-conjugated, BD Biosciences Pharmingen), CD86 (GL1, PE-conjugated, BD Biosciences Pharmingen), DC markers: CD11c (HL3, PE-conjugated, BD Biosciences Pharmingen), DEC-205 (NLDC-145, PE-conjugated, Cedarlane Labs, Ontario, Canada), a macrophage marker: F4/80 (CI:A3-1, PE-conjugated, Serotec, Oxford, UK); a B-cell differentiation antigen: CD19 (6D5, PE-conjugated, Southern Biotechnology, Birmingham, AL). Immunostaining was conducted as previously described [20,23,24], and flow cytometry was performed using a FACSCalibur (Becton, Dickinson and Company, NJ). Fluorescence data are expressed as the percentage of positive cells. Finally, the number of positive cells was calculated and expressed for each group (n = 8 in each group).

### In vitro studies

#### Preparation of bone marrow cells and splenocytes

Mice were anesthetized with sodium pentobarbital and exsanguinated from the cut abdominal aorta and vein. After removing the surrounding muscle tissue by rubbing with kleenex^® ^tissues, the bones were left in 70% ethanol for 3 min and washed with Dulbecco's calcium and PBS. Both ends of the bones were cut and then the marrow was flushed with PBS using a syringe with a 25G needle. The marrow suspension was passed through nylon mesh to remove small pieces of bone and debris and red blood cells were lysed with ammonium chloride. The spleen was pushed through a sterile stainless wire mesh (200 mesh) and red blood cells were also lysed with ammonium chloride. The cells were centrifuged at 400 × g for 5 min at 20°C. After washing twice with PBS, the cells were resuspended in the culture medium R10, which was GIBCO RPMI 1640 medium (Invitrogen Co.) supplemented with 10% heat-inactivated fetal bovine serum (FBS; MP Biomedicals Inc., Eschwege, Germany), 100 U/ml penicillin, 100 μg/ml streptomycin (Sigma Chemical), and 50 μM 2-mercaptoethanol (Invitrogen Co.). The numbers of viable cells were determined by the trypan blue (Invitrogen Co.) exclusion method.

#### Differentiation of BMDCs

BMDCs were differentiated using a modified protocol of Lutz et al [25]. In brief, bone marrow cells (4 × 10^5^/ml) were cultured in R10 medium containing 20 ng/ml recombinant mouse GM-CSF (Sigma Chemical) at 37°C in a 5% CO_2_/95% air atmosphere. On day 3, another same volume of medium was added to the culture. At day 6 of culture, half of the culture medium was poured into fresh R10 medium containing 20 ng/ml GM-CSF. At day 8 of culture, non-adherent and loosely adherent cells were collected by gentle pipetting, and centrifuged at 400 × g for 5 min at 20°C. These cells (immature BMDC) were resuspended in the medium. The numbers of viable cells were determined by the trypan blue exclusion method.

#### Exposure to CSBG in vitro

CSBG was dissolved in PBS. BMDCs were differentiated by culture with GM-CSF as described above. Differentiated BMDCs (1 × 10^6^/ml) on day 8 of culture with GM-CSF (20 ng/ml) were exposed to CSBG (1.25–12.5 μg/ml) or 0.1% DMSO (control) in the presence of GM-CSF (10 ng/ml) for 24 h. Thereafter, the phenotypes and function of BMDCs were evaluated in terms of the expression pattern of surface markers and antigen-presenting activity. On the other hand, splenocytes (1 × 10^6^/ml) were exposed to CSBG (1.56–12.5 μg/ml) or 0.1% DMSO (control) in the presence or absence of 100 μg/ml OVA for 72 h, and then the cell.

#### FACS analysis

For FACS analysis, the following monoclonal antibodies were used: I-A/I-E, CD80, CD86, CD11c, and DEC-205. FACS analysis was performed using the same protocol as in the *in vivo *study. This experiment was repeated four times using two to three animals in each group.

#### Evaluation of antigen-presenting activity of BMDCs

CSBG-exposed BMDCs were treated with 50 μg/ml mitomycin C (Kyowa Hakko Kogyo, Tokyo, Japan) for 20 min in a water bath at 37°C. Responder T cells were derived from a pool of splenocytes from OVA-sensitized ICR mice (three mice/each experiment). Briefly, mice were immunized with 40 μg OVA and 1 mg Al(OH)_3 _in 0.1 ml of saline given intraperitoneally, and splenocytes were harvested on day 14. T cells were isolated from the splenocytes using a nylon fiber column (Wako Pure Chemical Industries). Thereafter, OVA-sensitized T cells (2 × 10^5^) were co-cultured with BMDCs (5 × 10^3^) in the presence of 20 μg of OVA in 200 μl of R10 medium in 96-well flat-bottom plates. The co-culture of BMDCs and T cells was performed in triplicate at 37°C in a 5% CO_2_/95% air atmosphere. After 91 h, cell proliferation and cytokine production from T cells were measured. The same experiment was repeated three times using three animals per group.

#### Measurement of cell proliferation

Cell proliferation was measured with a Cell-Proliferation-ELISA Kit (Roche Molecular Biochemicals, Mannheim, Germany) according to the manufacturer's instructions. This technique is based on the incorporation of the pyrimidine analogue 5-bromo-2'-deoxyuridine (BrdU) instead of thymidine into the DNA of proliferating cells. BrdU incorporated into DNA is measured by a sandwich-type enzyme immunoassay using monoclonal anti-BrdU antibodies. Cell proliferation was measured by adding BrdU to each well 20 h before measurement. Absorbance of the samples was measured in an ELISA plate reader at a wavelength of 450 nm.

### Statistical analysis

Data are reported as the mean ± SE using Statview version 4.0 (Abacus Concepts, Inc., Berkeley, CA). To examine each (CSBG and OVA) factor's effect and interaction in the combination, two-way ANOVA was performed for *in vivo *results. Regarding *in vitro *studies, one-way ANOVA was applied. Bonferroni corrections were used for post hoc tests to examine the difference between the two groups. Significance was assigned to P-values smaller than 0.05.

## Results

### Effects of pulmonary exposure to CSBG on lung inflammation in the presence or absence of OVA

**Figure 1:**
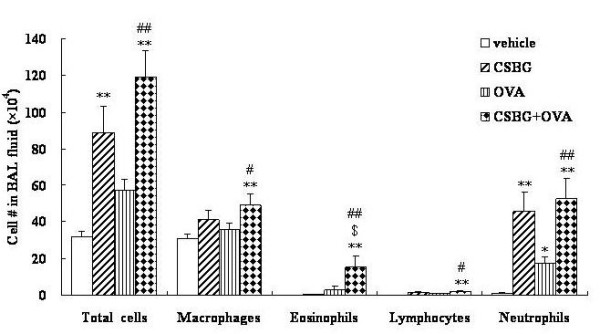
**Cellularity in bronchoalveolar lavage (BAL) fluid after intratracheal challenge**. Four groups of ICR mice were intratracheally administered vehicle, soluble β-glucan from *Candida albicans *(CSBG), ovalbumin (OVA), or CSBG + OVA for 4 wk. Bronchoalveolar lavage (BAL) was conducted 24 h after the last intratracheal instillation. Total cell counts were determined on fresh BAL fluid, and differential cell counts were assessed with Diff-Quik-staining. The results are presented as the mean ± SE (n = 8 in each group). *P < 0.05 and **P < 0.01 vs. vehicle; ^# ^P < 0.05 and ^## ^P < 0.01 vs. OVA; ^$ ^P < 0.01 vs. CSBG.

We investigated the cellular profile of BAL fluid and exhibited representative data (25 μg of CSBG used: Fig. 1). The number of total cells was significantly greater in the CSBG or CSBG + OVA group than in the vehicle group (P < 0.01). The number was significantly greater in the CSBG + OVA group than in the OVA group (P < 0.01). Exposure to CSBG + OVA increased the number of eosinophils as compared with vehicle exposure (P < 0.01). The number was significantly greater in the CSBG + OVA group than in the CSBG or OVA group (P < 0.01), and synergistic interaction between the two stimulators was noted (P = 0.02). The number of lymphocytes was significantly greater in the CSBG + OVA group than in the vehicle group (P < 0.01). The number was greater in the CSBG + OVA group than in the OVA group (P < 0.05). The number of neutrophils was significantly greater in the CSBG (P < 0.01), OVA (P < 0.05), or CSBG + OVA (P < 0.01) group than in the vehicle group. The number was significantly higher in the CSBG + OVA group than in the OVA group (P < 0.01). The number of macrophages was greater in the CSBG + OVA group than in the vehicle group (P < 0.01). The number was further significantly greater in the CSBG + OVA group than in the OVA group (P < 0.05). On the other hand, in experiments using 12.5 μg of CSBG, the data are similar as that using 25 μg, although the significance did not show profoundly (data not shown).

### Effects of CSBG on histological changes in the lung in the presence or absence of OVA

**Figure 2:**
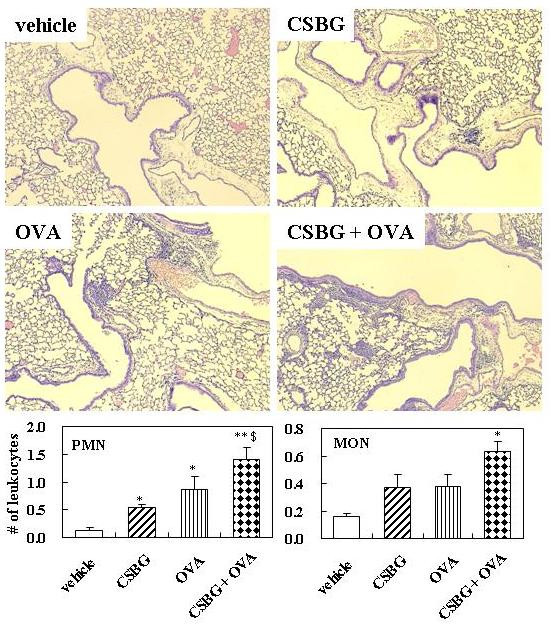
**Histological findings of hematoxylin and eosin (H&E)-stained lungs**. The 4 groups of mice were intratracheally inoculated with vehicle, CSBG, OVA, or CSBG + OVA for 4 wk. Lungs were obtained 24 h after the final intratracheal administration, and representative photographs of H&E-stained lung tissues are shown. Original magnification ×100. Polymorphonuclear leukocyte (PMN) and mononuclear cell (MON) infiltration is expressed as the average number of PMNs and MONs per millimeter of basement membrane. Values represent the mean ± SE (n = 5 in each group). * P < 0.01 vs. vehicle; ^$ ^P < 0.05 vs. CSBG.

We evaluated lung specimens stained with H&E 24 h after the final intratracheal instillation (Fig. 2). No pathological change was noted in lungs obtained from the vehicle group. The infiltration of neutrophils was moderate in the lungs from the CSBG and modest in those from the OVA group. On the other hand, the infiltration of eosinophils was moderate in lungs from the OVA group. Combined treatment with CSBG + OVA appeared to worsen PMN and MON sequestration into the lung parenchyma, as compared with OVA treatment alone.

To quantify the infiltration of inflammatory leukocytes around the airways, we expressed the number of these cells per length of basement membrane of the airways. The number of PMNs was greater in the CSBG (P < 0.05), OVA (P < 0.05), or CSBG + OVA (P < 0.01) group than in the vehicle group. The number was also significantly greater in the CSBG + OVA group than in the CSBG group (P < 0.05). The number of MONs was larger in the CSBG + OVA group than in the vehicle group (P < 0.05).

### CSBG exacerbates OVA-induced mucus metaplasia

**Figure 3:**
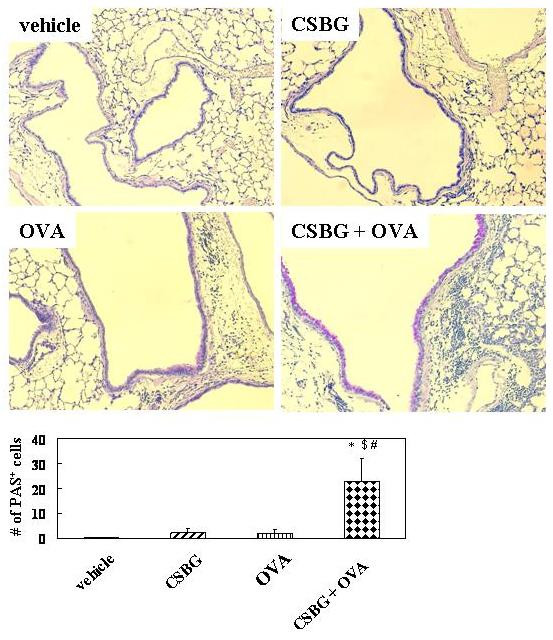
**Histological findings of the periodic acid Schiff (PAS)-stained lungs**. The 4 groups of mice were intratracheally inoculated with vehicle, CSBG, OVA, or CSBG + OVA for 4 wk. Lungs were obtained 24 h after the final intratracheal administration and representative photographs of PAS-stained lung tissues are shown. Original magnification × 200. Mucus cell hyperplasia in the bronchial epithelium is expressed as the average number of PAS-positive cells per millimeter of basement membrane. Values represent the mean ± SE (n = 5 in each group). * P < 0.01 vs. vehicle; ^# ^P < 0.01 vs. OVA; ^$ ^P < 0.01 vs. CSBG.

To evaluate airway epithelial injury and mucus hypersecretion, lung sections were stained with PAS (Fig. 3). CSBG or OVA alone induced modest mucus cell hyperplasia in the airway compared with the vehicle. However, the phenomenon markedly progressed in the CSBG + OVA group compared to the other groups. Semi-quantitative analyses also showed that CSBG or OVA alone modestly increased the number of mucus cells as compared with the vehicle. On the other hand, the number was significantly greater in the CSBG + OVA group than in the vehicle, CSBG, or OVA group (P < 0.01), and synergistic interaction between the two stimulators was noted (P = 0.02).

### Effects of CSBG on the lung expression of cytokines and chemokines in the presence or absence of OVA

**Figure 4:**
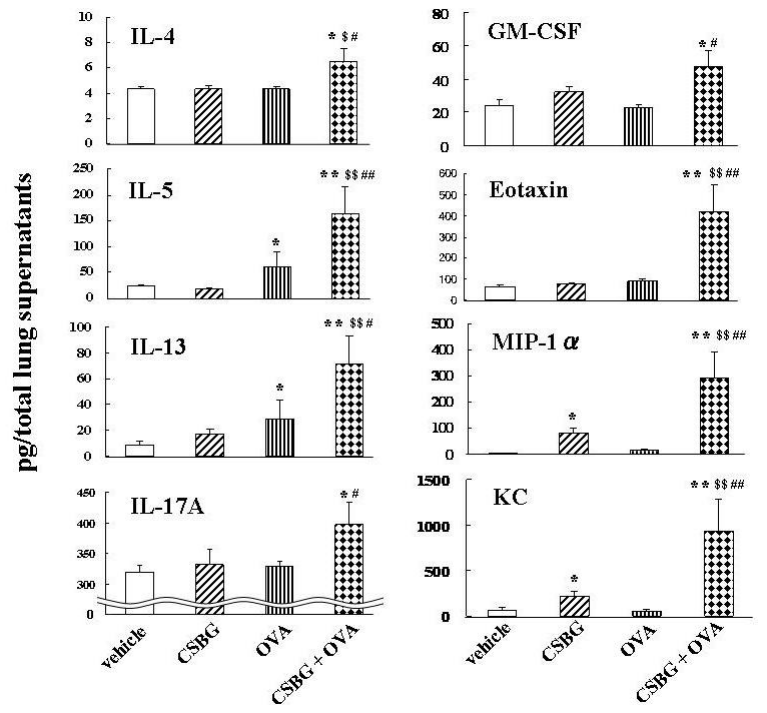
**Protein levels of cytokines and chemokines in the lung homogenates**. The 4 groups of mice were intratracheally inoculated with vehicle, CSBG, OVA, or CSBG + OVA for 4 wk. Lungs were removed and frozen 24 h after the last intratracheal administration. Protein levels of interleukin (IL)-4, IL-5, IL-13, IL-17A, granulocyte/macrophage-colony-stimulating factor (GM-CSF), eotaxin, macrophage inflammatory protein (MIP)-1α (C), and keratinocyte-derived chemoattractant (KC) in the lung homogenate supernatants were analyzed using ELISA. The results are presented as the mean ± SE (n = 8 in each group). * P < 0.05 and ** P < 0.01 vs. vehicle; ^# ^P < 0.05 and ^## ^P < 0.01 vs. OVA; ^$ ^P < 0.05 and ^$$ ^P < 0.01 vs. CSBG.

We quantified protein levels of cytokines such as IL-4, IL-5, IL-13, IFN-γ, IL-17A, and GM-CSF,, and chemokines such as eotaxin, MIP-1α, and KC related to allergic inflammation in the lung tissue homogenates (Fig. 4). Pulmonary exposure to CSBG elevated the levels of MIP-1α and KC compared to vehicle exposure (P < 0.05). OVA elevated the levels of IL-5 and IL-13 as compared to vehicle exposure (P < 0.05). On the other hand, CSBG plus OVA markedly increased the levels of all cytokines and chemokines compared to the vehicle (P < 0.05 for IL-4, IL-17A, and GM-SCF; P < 0.01 for IL-5, IL-13, eotaxin, MIP-1α, and KC), CSBG (P < 0.05 for IL-4; P < 0.01 for IL-5, IL-13, eotaxin, MIP-1α, and KC), or OVA (P < 0.05 for IL-4, IL-13, IL-17A, and GM-CSF; P < 0.01 for IL-5, eotaxin, MIP-1α, and KC). Furthermore, two-way ANOVA revealed synergistic interactions between CSBG and OVA on IL-4, IL-5, IL-13, IL-17A, eotaxin, MIP-1α, and KC (P < 0.05). IFN-γ level was not different between the experimental groups (data not shown).

### Effects of CSBG on the production of Igs in the presence or absence of OVA

**Figure 5:**
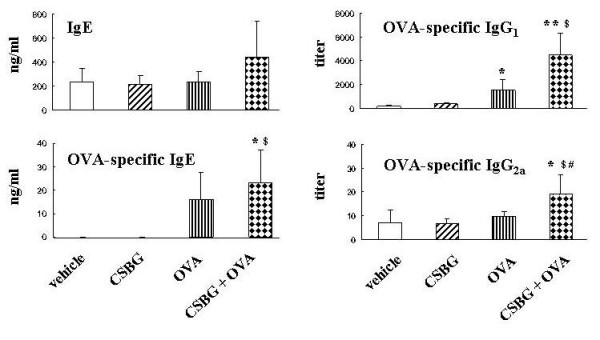
**Values of total IgE and OVA-specific IgG_1_, IgG_2a_, and IgE**. The 4 groups of mice were intratracheally administered vehicle, CSBG, OVA, or a combination of CSBG + OVA for 4 wk. Serum samples were retrieved 24 h after the last intratracheal instillation. Total IgE and OVA-specific IgG_1_, IgG_2a_, and IgE were analyzed using ELISA. The results are expressed as the mean ± SE (n = 13 in each group). * P < 0.05 and ** P < 0.01 vs. vehicle; ^# ^P < 0.01 vs. OVA; ^$ ^P < 0.01 vs. CSBG.

We measured total IgE and OVA-specific IgG_1_, IgE, and IgG_2a _levels (Fig. 5). The total IgE level was greater in the CSBG + OVA group than in the other groups (N. S.). The OVA-specific IgG_1 _titer was higher in the OVA (P < 0.05) or CSBG + OVA (P < 0.01) group than in the vehicle group. The titer was further greater in the CSBG + OVA group than in the CSBG group (P < 0.05). OVA-specific IgE concentration was greater in the CSBG + OVA group than in the vehicle group (P < 0.05). The value was also greater in the CSBG + OVA group than in the CSBG group (P < 0.05). The OVA-specific IgG_2a _titer was higher only in the CSBG + OVA group than in other groups (P < 0.05).

### Effects of in vivo exposure to CSBG on the expression of MHC class II with or without costimulatory molecules and APC-related markers in lung cells in the presence or absence of OVA

**Figure 6:**
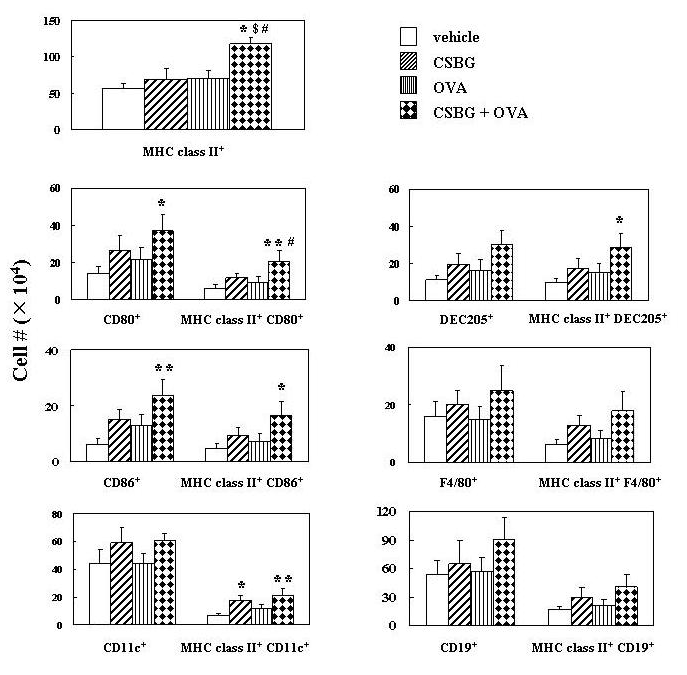
**The number of cells expressing MHC class II, co-stimulatory molecules, and leukocyte markers in the lung**. The 4 groups of mice were intratracheally administered vehicle, CSBG, OVA, or a combination of CSBG + OVA for 4 wk. Lung cells were prepared 4 h after the last intratracheal instillation. The expression of cell surface molecules was analyzed by flow cytometry. The numbers of each cell type in the lung are shown. Values are presented as the mean ± SE (n = 8 in each group). * P < 0.05 and ** P < 0.01 vs. vehicle; ^# ^P < 0.05 vs. OVA; ^$ ^P < 0.05 vs. CSBG.

The number of MHC class II^+ ^cells was greater in the CSBG + OVA group than in the other groups (P < 0.05)(Fig. 6). Also, a two-way ANOVA revealed an interaction between CSBG and OVA on the number (P = 0.04). The number of CD80^+ ^or CD86^+ ^cells was greater in the CSBG + OVA group than in the vehicle group (P < 0.05 for CD80^+^, P < 0.01 for CD86^+^). Furthermore, the number of MHC class II^+ ^CD80^+ ^or MHC class II^+ ^CD86^+ ^cells was also greater in the CSBG + OVA group than in the vehicle group (P < 0.05 for MHC class II^+ ^CD86^+^, P < 0.01 for MHC class II^+ ^CD80^+^). The number of MHC class II^+ ^CD80^+ ^was further greater in the CSBG + OVA group than in the OVA group (P < 0.05). In addition, a two-way ANOVA revealed an interaction between CSBG and OVA on the number of MHC class II^+ ^CD80^+ ^(P = 0.045). The number of MHC class II^+ ^CD11c^+ ^cells was higher in the CSBG (P < 0.05) or the CSBG + OVA (P < 0.01) group than in the vehicle group.

The number of MHC class II^+ ^DEC205^+ ^cells was greater in the CSBG + OVA group than in the vehicle group (P < 0.05). On the other hand, the number of F4/80^+ ^or CD19^+ ^cells with or without MHC class II^+ ^was not significantly different among the experimental groups.

### Effects of CSBG on splenocyte proliferation in vitro

**Figure 7:**
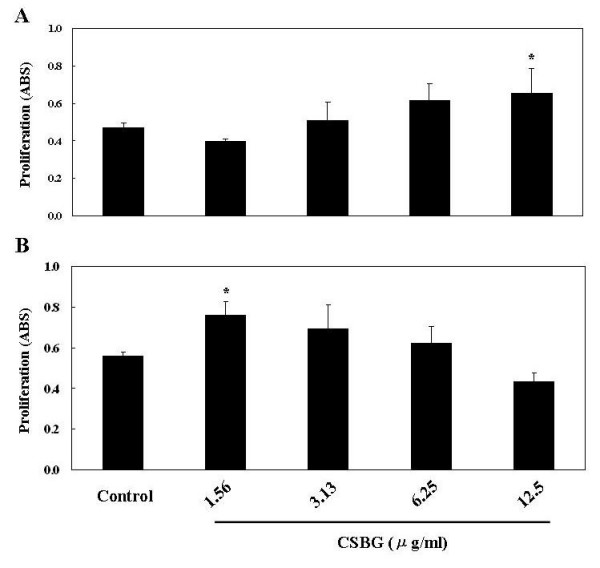
**Splenocyte proliferation**. Splenocytes (1 × 10^6^/ml) from naïve ICR mice were exposed to CSBG (1.56–12.5 μg/ml) or 0.1% dimethyl sulfoxide (control) in the absence (A) or presence (B) of OVA (100 μg/ml) in 200 μl of R10 medium for 72 h. Cell proliferation of splenocytes was evaluated by ELISA. Data represent the mean ± SE of three animals from one experiment representative of three experiments. * P < 0.05 vs. control.

The proliferation of splenocytes was examined after exposure to CSBG or DMSO (control) in the presence or absence of OVA for 72 h (Fig. 7). Exposure to CSBG increased splenocyte proliferation in the absence of OVA in a dose-dependent manner showing an overall trend, with significance at a dose of 12.5 μg/ml (P < 0.05: Fig. 7A). Also, CSBG increased it in the presence of OVA in a dose-independent manner (maximal activity at 1.56 μg/ml: P < 0.05: Fig. 7B).

### Action of CSBG on the expression of surface molecules on BMDCs in vitro

**Figure 8:**
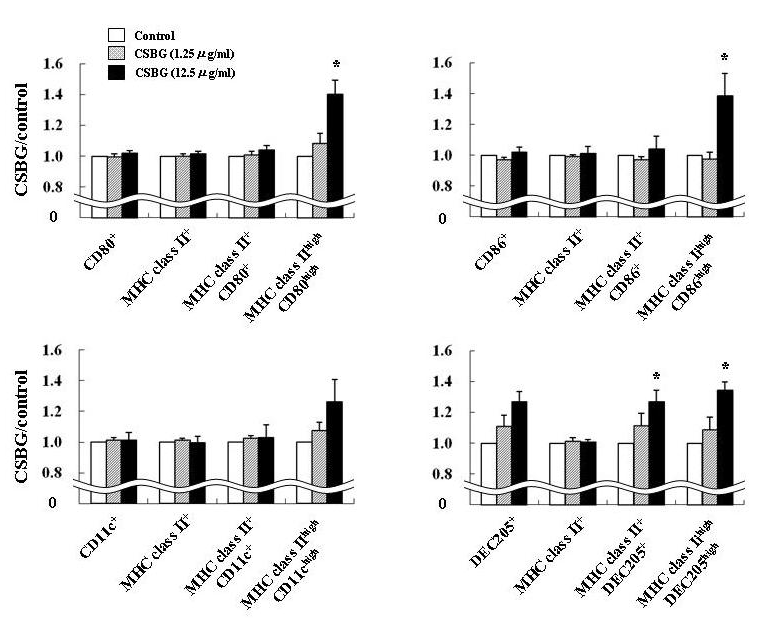
**Surface expression of molecules related to antigen-presentation on bone marrow-derived dendritic cells (BMDCs)**. Immature BMDCs from ICR mice were exposed to CSBG as described in Materials and Methods. After exposure, the expression of molecules related to antigen-presentation was analyzed by flow cytometry. The percentages of each cell type in the BMDC population are shown. Data represent the mean ± SE of three animals from one experiment, representative of three experiments. * P < 0.05 vs. corresponding control.

Immature BMDCs were exposed to CSBG for 24 h. The surface expression of MHC class II, CD80, CD86, CD11c, and DEC205 (Fig. 8) on cells was analyzed to estimate the maturation/activation of BMDCs. The percentage of CD80^+^, CD11c^+^, DEC205^+^, MHC class II^+^CD80^+^, MHC class II^+ ^CD11c^+^, or MHC class II^+ ^DEC205^+ ^cells was increased by the addition of CSBG in a dose-dependent fashion with overall trend. Of note, the increase was profoundly in the ratio of double highly positive cells (MHC class II^high^CD80^high^, MHC class II^high^CD86^high^, and MHC class II^high^DEC205^high^) with significance at its high dose (12.5 μg/ml)(P < 0.05) as compared to control.

### Effect of CSBG on the OVA-specific syngeneic T-cell-stimulating capacity of BMDCs in vitro

**Figure 9:**
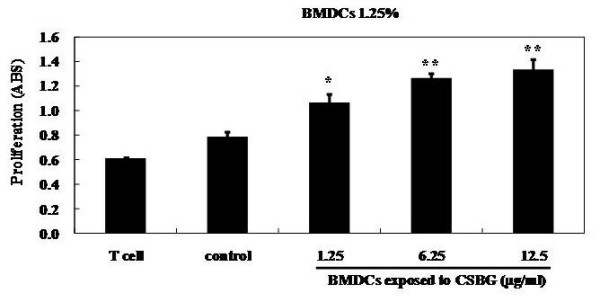
**OVA-specific syngeneic T-cell-stimulating capacity of BMDCs**. Immature BMDCs from ICR mice were exposed to CSBG in the same manner as shown in Figure 8. Splenic T cells (2 × 10^5^) from OVA-sensitized ICR mice were co-cultured with BMDCs (5 × 10^3 ^for 1.25%) from mice in the presence of OVA (20 μg) in 200 μl of R10 medium for 4 d. Thereafter, T-cell proliferation was evaluated. Data represent the mean ± SE of three animals from one experiment, representative of three experiments. *P < 0.05 and **P < 0.01 vs. control.

BMDC function was assessed via their capacity to stimulate antigen-specific syngeneic T-cell proliferation (Fig. 9). The proliferation of T cells (responder cells) was increased by the addition of BMDCs (stimulator cells). The reaction was significantly increased when BMDCs were exposed to more than 1.25 μg/ml of CSBG. Furthermore, Th cytokine (IL-4 and IFN-γ) levels in culture supernatants were measured by ELISA. Although the protein levels were increased by CSBG exposure dose-independently, the difference did not reach statistical significance (data not shown).

## Discussion

The present study has demonstrated that repetitive pulmonary exposure to CSBG potentiates OVA-related eosinophilic airway inflammation with mucus metaplasia, which is concomitant with the increased local expression of proinflammatory cytokines including IL-4, IL-5, IL-13, IL-17A, eotaxin, MIP-1α, and KC. Also, exposure to CSBG plus allergen increases the numbers of MHC class II^+ ^cells and those expressing MHC class II and CD80 (a co-stimulatory molecule) in the lung as compared to that of others *in vivo*. Finally, CSBG increases splenocyte proliferation and partially promotes the phenotypic and functional activation of BMDCs *in vitro*.

Correlation between fungi and allergic responses has been implicated. Airborne fungi and their products may contribute to the development and exacerbation of allergic airway diseases [26,27]. Increased spore counts and fungal allergen levels correlate with allergic symptoms [28-30]. Consistent with these epidemiological reports, fungal products including proteins reportedly induce immunologic and proinflammatory reactions and consequent Th2-related responses and the destruction of mucosal barrier functions [31-33]. β-glucan from both outdoor (*Cladosporium herbarum*) and indoor (*Penicillium chrysogenum*) fungi and from baker's yeast exhibit adjuvant properties for Th2 response related to OVA *in vivo *[8]. Further, peripheral blood mononuclear cells (PBMCs) from asthma patients produced significantly higher IL-5 protein levels on stimulation with *C. albicans *than those from non-allergic patients *in vitro *[34], implicating a role in the exacerbation of asthma. However, scientific evidence regarding the correlation between pathogenic fungi including *C. albicans *and allergic inflammation is not enough.

Furthermore, investigation of the immunoregulatory effects of cell wall components from fungi has been inadequate. Zymosan, a cell wall preparation mixed with β-glucan and mannan residues from the fungus *Saccharomyces cerevisiae*, is known to induce the production of proinflammatory cytokines and chemokines [35,36], and to activate the complement system via an alternative pathway [37,38]. Furthermore, zymosan can induce multiple types of immunopathological diseases, resembling human diseases, e.g., peritonitis [39] and arthritis [40], *in vivo*. It has been indicated, on the other hand, that β-glucan itself serves various biological activities such as triggering the activation of complement and the production of inflammatory mediators including leukotrienes and tumor necrosis factor-α [9,10,41]. However, it has not been fully addressed how β-glucan derived from clinically important pathogenic fungi modulates the immune response, particularly *in vivo*. Given the background, CSBG, isolated and solublized from *C. albicans*, may provide a hint for the impacts of nosocomial fungal infection on the induction/aggravation of allergic airway inflammation, particularly in the context of its responsible constituents.

In the present study, initially, we showed that repetitive intratracheal exposure to CSBG did not induce allergic airway response in terms of lung eosinophilia, the Th2 response, or mucous hyperplasia, suggesting that the component alone does not have potent Th2-related immunogenicity. Possible explanations for this unexpected phenomenon may include the *in vivo *experimental protocol such as exposure means, dose, and/or duration, and absence of adjuvant. In support of this, exposure to CSBG alone induced a moderate activation of BMDCs and lymphocytes directly (through immunological synapse)/indirectly *in vitro*. Moreover, we have previously found that a single intratracheal administration of CSBG can induce mucus hyperplasia, one of the consequences of the Th2 (IL-13)-dominant response and the enhanced phosphorylation of STAT6, one of the pivotal transcriptional factors in the Th2 milieu [42]. Thus, other protocols should be examined in future in order to clarify the effects of subacute/chronic exposure to CSBG on the induction of Th2 immunity with pathophysiology. On the other hand, CSBG alone induced airway neutrophilia, whose mechanisms remain unidentified. We and others have shown that *Candida *or CSBG can activate innate immunity to produce KC (IL-8), a potent neutrophilic chemoattractant [13,15,43]. Furthermore, CSBG has been shown to activate complement systems, including anaphylatoxin (C3a, C5a) release [44]. Accordingly, these mechanisms could be candidates. Alternate, the effects might be caused by other unknown factors co-extracted from *C. Albicans *other than endotoxin.

In turn, we showed that CSBG synergistically enhanced allergic airway inflammation induced by OVA. The CSBG plus OVA group exhibited significant augmentation of eosinophilic lung inflammation with concomitant amplification of the lung expression of Th2 cytokines and chemokines and related Ig productions in the circulation compared to the OVA group. In addition, phosphorylation of STAT6 in the lung was more potently found in the CSBG + OVA than in the other groups (evidenced by Western blot analysis: data not shown). Thus, it is likely that *Candida*-derived β-glucan can synergistically drive a Th2-skewed pathophysiology related to another allergen as a promoter, even if the dose is low enough to barely induce eosinophilic inflammation in the absence of other immunogenic allergen. However, why and how the macromolecule glucose polymers displayed adjuvant effects on the allergic condition when co-administered with relatively low molecular weight OVA must be resolved in the future.

The cellular target of CSBG in the exacerbation on allergic airway inflammation induced by OVA is currently unknown. Our first *in vitro *experiment implied that CSBG can induce immune cell (splenocyte) activation in the presence or absence of OVA. However, its detailed cellular contribution is not known, since splenocytes contain many immune cell populations such as macrophages/monocytes, dendritic cells, and lymphocytes. Antigen-presenting cells (APCs) are known to be important for both innate and adaptive immunity. APCs including DCs, macrophages, and B cells play essential roles in the pathogenesis of asthma [45-47]. In particular, DCs are recognized as professional APCs, exhibiting a potent antigen-presenting capacity for T cells [48]. β-glucan of *Candida *reportedly promotes the maturation of human monocyte-generated DCs [49]. Therefore, we evaluated the effects of CSBG on the phenotype and function of DCs *in vitro*. As a result, although CSBG did not significantly enhance the total expression of surface markers (DEC205, CD80, and CD86) as an activation marker and accessory molecules on BMDCs, the rate of highly expressed molecules was significantly greater in the higher concentration of CSBG than the control. On the other hand, CSBG exposure significantly amplified OVA-specific syngeneic T cell reactivity against BMDC. Further, the numbers of cells expressing MHC class II or MHC class II and CD80 in the lung were significantly greater in the CSBG + OVA group than in the other groups *in vivo*. Accordingly, the *in vitro *and *in vivo *data may lead to the scenario that CSBG can synergistically increase the number of APCs recruited into the lung in asthmatic mice, and, in turn, can amplify the functional activation of APCs including DCs, which confer synergism for the enhancement of asthma pathophysiology. This notion is also supported by the observation that the lung expression of GM-CSF was greater in the CSBG + OVA group than in the other groups, since GM-CSF is a critical cytokine for DC maturation/activation as applied in the present *in vitro *study.

Whether CSBG preferentially enhances the Th2 response remains obscure. In the present study, CSBG plus OVA amplified the production of OVA-specific IgG_2a _as compared to OVA alone *in vivo *and CSBG activated DC to produce/release higher levels of OVA-stimulated IFN-γ from T cells than control, implicating its adjuvant activity for Th1 immunity as well as Th2 immunity *in vivo *and partially *in vitro*. Furthermore, the lung level of IL-17A was significantly greater in the CSBG + OVA group than in the CSBG or OVA group. The *Candida *cell wall fraction reportedly exacerbates collagen-induced arthritis, which is concomitant with the elevation of IFN-γ and bovine type II collagen-specific IgG_2a _[50]. On the other hand, *C. albicans *extract induces IL-5 production from PBMCs in asthmatic patients *in vitro *[51]. In addition, Th17 cells were recently implicated in host immunity against *Candida *infection [52] as well as allergic inflammation [53]. Thus, it may be proposed that CSBG can induce both Th1- and Th2-, and even Th17-mediated immunopathology, whose dominance may depend on the study designs (*in vivo*, *in vitro*), tissues (lung, joint)/cells (isolated/generated from splenocytes, PBMCs), and/or the driving mode (Th1/Th2/Th17 conditions). Otherwise, from our present results, it is likely that CSBG disrupts Th immunity, especially under pathological Th conditions, *in vivo*.

## Conclusion

In summary, repetitive pulmonary exposure to CSBG facilitated allergic lung inflammation, at least partly, via the enhanced expression of Th cytokines. As for possible cellular mechanisms, these CSBG's effects were correlated with the phenotypic and functional activation of APCs including DCs, and partly with the migration/infiltration of these cell populations in the inflamed tissues. These results provide evidence for pathogenesis of asthma development and/or exacerbation due to (biological and residence) environmental factors.

## Abbreviations

(CSBG): soluble β-glucan from *C. albicans*; (STAT): signal transducer and activator of transcription; (OVA): ovalbumin; (BMDC): bone marrow-derived dendritic cell; (OX-CA): NaClO-oxidized candida; (DMSO): dimethyl sulfoxide; (PBS): phosphate-buffered saline; (BAL): bronchoalveolar lavage; (H&E): hematoxylin and eosin; (PAS): periodic acid-Schiff; (PMN): polymorphonuclear leukocyte; (MON): mononuclear cell; (IL): interleukin; (IFN): interferon; (GM-CSF): granulocyte/macrophage-colony-stimulating factor; (MIP): macrophage inflammatory protein; (KC): keratinocyte-derived chemoattractant; (BSA): bovine serum albumin; (BrdU): 5-bromo-2'-deoxyuridine; (N. S.): not significant; (PBMC): peripheral blood mononuclear cell; (APC): antigen-presenting cell.

## Competing interests

The authors declare that they have no conflicting interests.

## Authors' contributions

KI performed all procedures of *in vivo *experiments and wrote the first draft of the manuscript. EK performed all procedures of *in vitro *experiments. RY participated in sample and data collection. TO and HTam contributed to the study design and synthesis of CSBG. YA, KI, and NO contributed to all aspects of study design. HTak assisted in data analysis and editing the manuscript. All authors have read and approved the final version of the manuscript.
